# Dogmas, challenges, and promises in phase III allergen immunotherapy studies^☆^

**DOI:** 10.1016/j.waojou.2021.100578

**Published:** 2021-09-28

**Authors:** Pieter-Jan De Kam, Matthias F. Kramer, Mohamed H. Shamji, Kemi Oluwayi, Matthew D. Heath, Erika Jensen-Jarolim, Markus H. Berger, Uwe E. Berger, Anke Graessel, Fiona Sellwood, Stefan Zielen, Christian Vogelberg, Petra Zieglmayer, Ralph Mösges, Ludger Klimek, Lawrence M. DuBuske, Wayne G. Shreffler, Jonathan A. Bernstein, Thomas M. Kündig, Murray A. Skinner

**Affiliations:** a Allergy Therapeutics (UK) Ltd, Worthing, United Kingdom; b Bencard Allergie GmbH, Munich, Germany; c Immunomodulation and Tolerance Group, Allergy and Clinical Immunology, Inflammation, Repair & Development, MRC Asthma UK Centre Imperial College London, London, United Kingdom; d Institute of Pathophysiology and Allergy Research, Center of Pathophysiology, Infectiology and Immunology, Medical University of Vienna, Vienna, Austria; e The Interuniversity Messerli Research Institute, Univ of Veterinary Medicine Vienna, Medical University Vienna, University Vienna, Austria; f Aerobiology and Pollen Information Research Unit, Department of Oto-Rhino-Laryngology, Medical University of Vienna, Vienna, Austria; g Department for Children and Adolescents, Division of Allergology, Pulmonology and Cystic Fibrosis, Goethe University, Frankfurt, Germany; h Department of Paediatrics, Universitätsklinikum Carl Gustav Carus, Technical University Dresden, Germany; i Vienna Challenge Chamber, Vienna, Austria; j Karl Landsteiner University, Krems, Austria; k Institute of Medical Statistics and Computational Biology, Faculty of Medicine, University of Cologne, Cologne, Germany and ClinCompetence Cologne GmbH, Cologne, Germany; l Center for Rhinology and Allergology, Wiesbaden, Germany; m Division of Allergy and Immunology, Department of Internal Medicine at George Washington University Medical Faculty Associates in Washington, Washington, DC, United States; n Massachusetts General Hospital and Harvard Medical School, Boston, MA, United States; o Department of Internal Medicine, University of Cincinnati College of Medicine, and Partner of Bernstein Allergy Group and Bernstein Clinical Research Center, Cincinnati, OH, United States; p Dermatology, University Hospital Zurich, Zurich, Switzerland

**Keywords:** Allergen immunotherapy, Phase III, Placebo effect, Biomarker, Blinding

## Abstract

The concept of treatment of an allergy with the offending allergen was introduced more than a century ago. Allergen immunotherapy (AIT) is the only disease modifying treatment of allergic diseases caused by inhalational allergens and insect venoms. Despite this, only few AIT products have reached licensure in the US or an official marketing authorization status in European countries. Moreover, most of these AIT products are provided on an individual patient basis as named patient products (NPP) in Europe, while individualized preparations of (mixed) allergenic extract vials for subcutaneous administration (compounding) is common practice in the US. AIT products are generally considered safe and well tolerated, but the major practical clinical development challenge is to define the optimal dose and prove the efficacy and safety of these products using state-of-the art Phase II and pivotal Phase III studies. In planning Phase II-III AIT studies, a thorough understanding of the study challenges is essential (e.g. variability and non-validated status of subjective primary endpoints, limitations of pollen season definitions) and dogmas of these products (e.g., for sublingual immunotherapy (SLIT) trials double-blinding conditions cannot be maintained, resulting in stronger placebo responses in the active treatment group and inflated treatment effects in Phase III). There is future promise for more objective biomarker endpoints (e.g. basophil activation (CD63 and CD203c), subsets of regulatory dendritic, T and B cells, IL-10–producing group 2 innate lymphoid cells; alone or in combination) to overcome several of these dogmas and challenges; innovation in AIT clinical trials can only progress with integral biomarker research to complement the traditional endpoints in Phase II-III clinical development. The aim of this paper is to provide an overview of these dogmas, challenges and recommendations based on published data, to facilitate the design of Phase III studies and improve the evidence basis of safe and effective AIT products.

## Introduction

Allergen immunotherapy (AIT) (or hyposensitization) was first described by Dunbar 1903 as a “passive vaccination”.^1^ However, it is commonly attributed to Noon and Freeman who transferred the concept to an active vaccination back in 1911: the practice of administration of increasing amounts of an allergen extract to a sensitized subject to subsequently reduce the symptoms induced by natural exposure to the causative allergen without eliciting anaphylactic reactions to increasing doses.^2^ This concept of desensitization has been widely used for the past century; immunotherapy is considered as the only immunomodulating treatment for immunoglobulin E (IgE) mediated allergic disorders induced by allergens.

The regulatory history of AIT products is much shorter. For several decades, AIT products were not recognized as medicinal products that should be subject to any standard legislation. Many AIT products were marketed as Named Patient Products (NPPs) in various European countries and individually produced for every single patient. These NPPs often consist of mixes of various allergens, sometimes with little rationale on combination effects of allergens from different species and different source materials, which is nowadays only applicable for rare allergens. The first AIT marketing authorization applications (MAA) in European and other countries (eg, Canada) were granted for more common allergens in the early 1970s based on limited clinical trial data, and these products are currently still available as marketed products. Over the last two decades, the international regulatory framework for AIT has undergone multiple changes. In 1996 a European Medicine Agency (EMA) “Note for guidance on allergen products” was released (CPMB/BWP/243/96) followed in 2001 by a Directive lifting therapy and diagnostic allergens to medicinal products (2001/83). In 2008, the Paul-Ehrlich-Institute (PEI) was the first regulatory agency to release legislation to require marketing authorization (MA) dossiers for more common AIT products provided as NPPs in Germany. In 2009, EMA introduced the guideline on the clinical development of products for specific immunotherapy for the treatment of allergic diseases.^3^ Since the introduction of the Therapy Allergen Ordinance (TAO) in Germany, over 6400 NPPs have been removed from the German market and of the TAO applications submitted in 2010, only 65 (50%) were still active in 2019^4^ Furthermore, other European regulators are introducing similar initiatives (eg, AIFA in Italy) and recently the new Pan-European initiative (CMDh/399/2019) has been introduced to further reduce the use of NPPs at the European level. The US Federal Drug Administration (FDA) has released various non-binding guidance documents describing FDA's current thinking on various regulatory topics related to development of AIT products. In practice, FDA reviewers closely collaborate with Investigational New Drug (IND) sponsors using various well-defined meetings (eg, Pre-IND meeting, end-of-Phase II meeting) to agree on the safety and efficacy requirements of the AIT product, and to ensure quality of the manufacturing process.

While for FDA a Paediatric Study Plan needs to be submitted within 60 days of the End-of-Phase II meeting, a one-year paediatric study to be conducted after Biologics License Application is generally sufficient, the EMA have raised the paediatric regulatory hurdle for paediatric development of AIT products. The EMA paediatric committee (PDCO) requires a long-term paediatric and adult Phase III study (3 years on double-blind, placebo-controlled therapy and 2 years follow-up) for the first AIT product completing a short-term pivotal Phase III study in adults. Moreover, EMA has introduced a paediatric compliance check to require the long-term paediatric study of the selected product to be started before the first MAA for short term treatment in adults can be evaluated, which also requires the parallel start of a long-term study in adults of the selected product.^5^ This highly increased paediatric regulatory burden for AIT products imposed by EMA will affect the AIT market and treatment options available in the future for patients with allergic rhinitis/conjunctivitis, and could create a long-term shortage of availability of some of the best-characterized products in the European Union.

This Position Paper aims to discuss the major challenges for Phase III clinical development of aeroallergen AIT products and to provide recommendations to guide a successful MA of such AIT products, in consideration of these regulatory guidelines. The challenges of pivotal Phase III AIT studies can be summarized as follows:

(i) Imperfect correlation between the Phase II surrogate endpoint results (ie, provocation test and exposure chamber endpoints) to the field study endpoint in Phase III
(ii) Definition, non-validation status, and substantial variability of the European Academy of Allergy and Clinical Immunology (EAACI) recommended primary endpoint for pivotal Phase III studies: The combined symptom and medication score (CSMS)
(iii) Placebo effects, blinding conditions in AIT and their influence on Phase III study outcomes
(iv) Placebo composition: active or inactive ingredients, which can potentially bias AIT treatment effects in Phase III
(v) Variability in allergen exposure and relation to symptom and medication scores and treatment effects
(vi) Patient selection and heterogeneity

We aim to discuss the challenges of evaluating AIT products in studies that conform to dogmas of clinical trial design and provide recommendations for designing Phase 3 studies that are needed to support the development of AIT products that are safe and effective.

## Challenges

### Imperfect correlation between the phase II surrogate endpoint results to the field study endpoint in phase III

The aim of Phase II AIT studies is to evaluate a dose-response relationship and to determine the optimal efficacious and safe dose to be used for Phase III. According to the EMA guidance, not only field studies can be used to establish a dose response in Phase II, but also surrogate efficacy endpoints such as provocation tests (eg, conjunctival, nasal, or bronchial provocation or allergen exposure in environmental exposure chambers [EEC]) may be used as primary end-points in Phase II.^3^ Of the proposed surrogate outcomes, the use of an EEC for Phase II studies has been most strongly encouraged by regulators, being considered as a potential alternative to field outcomes. However, several studies have shown that EEC and provocation studies have repeatedly over-estimated effect sizes observed in Phase III (Table 1).

**Table 1:** Overview of the most recent products for which Phase II have been performed with EEC and provocation tests and the pivotal Phase III study has been completed.

Route/Ref.	Allergen source	Allergen preparation	Model	Phase II result(s) End-point/main result	Phase III End-point/main results
SCIT^6^^,^^7^	Grass pollen	Peptide	CPT	25.6% improved thresholds by at least one concentration step compared to placebo (p = 0.023)	CSMS -15.5% (P = 0.041)
SCIT^8^^,^^9^	Cat	Peptide	Exposure chamber	28.3% improvement from placebo (p = 0.01)	Combined Score −1.3% (P = 0.439)
SCIT^10^^,^^11^	HDM	Peptide	CPT	Highest effect size −36.7% (p = 0.026)	Combined Score −4.2% (P = 0.26)
SCIT^12^	HDM	Allergoid	NPT	Highest effect size −48.1% (p < 0.0001)	CSMS -9.2% EudraCT2016-000051-27
SCIT^13^	Birch pollen	Allergoid with adjuvants MCT and MPL	CPT	Highest dose −32.3% (p < 0.001)	Phase III study completed in 2018 – PEI agreement that primary end-point was invalidated due to technical issues making it impossible to reconstruct primary end-point data
SLIT^a^,^14^^,^^15^	Birch pollen	Drops (non-modified)	NPT	Highest effect size −58.4% (p < 0.0001)	CSMS, - 32% (p < 0.0001)
SLIT^a^,^16^^,^^17^	HDM	Tablet	Exposure chamber	Highest effect size −48.6% (p < 0.001)	Total combined rhinitis score −18% (p = 0.01)

a Product has received marketing authorization status and/or US licensure; CPT = conjunctival provocation test; CSMS = combined symptom and medication score; HDM = house dust mite; NPT = nasal provocation test; PEI= Paul Ehrlich Institute; Ref. = Reference; SCIT = subcutaneous immunotherapy; SLIT = sublingual immunotherapy

Ideally, the dose-response in Phase II studies should be designed to show a dose-response plateau in the primary efficacy parameter, and the optimal dose is generally selected as the lowest dose reaching the plateau, assuming justifiable adverse effects with optimal risk-benefit-ratio. However, it is noteworthy that, except one of the birch Phase II studies,^13^ most of the successful Phase II studies listed in Table 1 failed to demonstrate a clear plateau in a dose-efficacy relationship. This indicates that the doses of several products which are in Phase III development and/or have reached MA status may not be fully optimized. Interestingly, these Phase II studies show a general trend towards several-fold higher optimal doses to be evaluated in Phase III than the doses used as NPP, although evidence of efficacy exists for NPP dose levels in some cases.^18^

Furthermore, remarkably, none of the recent subcutaneous immunotherapy (SCIT) products has a successful Phase III study reported so far, while several sublingual immunotherapy (SLIT) studies showed success in Phase III. This contrasts the historical results of well-powered, double-blind, randomized controlled trials versus placebo where SCIT is considered to show a more beneficial efficacy pattern than SLIT. This is evidenced by a Cochrane review from randomized, blind head-to-head comparisons, where SLIT products consistently showed less pronounced point estimates on both symptom and medication scores than subcutaneous products.^19^ More recent pivotal Phase III field studies usually require large sample sizes to demonstrate relatively small Phase III treatment effects of AIT. The recent largest SLIT Phase III study has enrolled 1607 patients (NCT02443805) to demonstrate only a 16.9% efficacy benefit of a mite tablet in Phase III.^20^

### Poor definition, non-validation status, and substantial variability of the primary end-point measure

The most frequently used scoring systems are the CSMS^24^ and total combined score (TCS).^25^ Both scores are very similar in composition, with the difference mainly in the calculation of the medication score (Table 2). Several other variants of combinations of symptoms scores and medication scores have been used and some have been published.^26^ The EMA and Food and Drug Administration (FDA) guidelines on the clinical development of allergic rhinitis and the World Allergy Organization (WAO) recommend a scoring combination of allergic symptoms and relief medications for pivotal Phase III studies.^21^, ^22^, ^23^ There are however, some clear disadvantages of using combinations of symptom scores and medication scores as a primary end-point in Phase III AIT studies. Firstly, from a statistical perspective, there are flaws in the methodology of composing these composite end-points, as it is highly questionable that equally weighing and combining symptom and medication scores provides the optimal endpoint choice. Ideally, symptom and medication questionnaires are first optimized and linguistically and psychometrically validated followed by optimization of weighing factors of individual symptom and medication scores using statistical simulation techniques and receiver operating characteristic (ROC) curves which are subsequently validated using available Phase III datasets. Moreover, the routinely used primary endpoints CSMS and TCS are also less sensitive in statistical testing due to the ordinal nature of the score.^27^ An extensive PubMed database search highlighted the limited validation of a symptom score without any study validating a medication score, which tend to be arbitrary insofar as the derivation of the scores attributed to specific reliever medication use.^28^ In addition, no minimal clinical important difference (MCID) has been established for any of the CSMSs. The WAO recommendation of a MCID of 20% has generally not been accepted by regulators.^23^ In previous studies, sample size calculations have been based on a MCID of 23% in grass allergy with limited rationale.^29^, ^30^, ^31^

**Table 2:** Composition of the most frequently used primary end-point scores for AIT Phase III studies: CSMS and TCS.

A) Symptom Score: CSMS + TCS	
**Conjunctival Symptoms**	Gritty^a^ feeling/itchy/red eyes
	Watery eyes
**Nasal Symptoms**	Blocked nose
	Runny nose
	Itchy nose
	Sneezing
**Each of the 6 symptoms will be scored using a 4-point severity scale** 0 = No symptoms 1 = Mild symptoms (sign/symptom clearly present, but minimal awareness; easily tolerated) 2 = Moderate symptoms (definite awareness of sign/symptom that is bothersome but tolerable) 3 = Severe symptoms (sign/symptom that is hard to tolerate; causes interference with activities of daily living and/or sleeping).	

B) Medication Score: Combined Symptom Medication Score (CSMS)		
Step	Relief medication	Score
	No relief medications used	0
1	Oral antihistamine/Ocular antihistamine	1
2	Intranasal corticosteroid with Step 1 medication(s)	2
3	Oral corticosteroids with Step 1 and Step 2 medications	3
**Maximum daily Medication Score (dMS)**		3

C) Medication Score: Total Combined Score (TCS)	
Relief medication	Score
No relief medications used	0
Oral antihistamine	Each tablet taken corresponds to a score of 6 with a maximum daily score of 6
Ocular antihistamine	Each drop corresponds to a score of 1.5 per eye with a maximum daily score of 6
Intranasal corticosteroid	Each spray corresponds to a score of 2 with a maximum daily score of 8
**Maximum daily Medication Score (dMS)**	20

a Gritty feeling is only applicable for the definition of TCS. CSMS = combined symptom medication score; TCS = total combined score

An attempt to validate the CSMS was undertaken using an anchor-based method in birch pollen allergy.^15^ This analysis showed a strong positive correlation between the post-intervention RQLQ-S scores and CSMS during the birch pollen season (r = 0.68 and p < 0.0001).^33^ It was calculated that a clinically relevant increase of 0.5 points improvement in RQLQ-S corresponds to a CSMS improvement of 21% (95% CI: 19–23%). A study to validate the CSMS (NCT03850626) has recently been completed, using established questionnaires as tools (RQLQ-S, Asthma Quality of Life Questionnaire, Asthma Control Test, Rhinitis Control Assessment Test and the Visual Analogue Scale). Nevertheless, it is unclear if such a non-interventional study with a limited sample size (n = 200) selecting a combination of grass, tree and mite allergic patients will indeed be able to support its objective.

An additional complication for the planning of pivotal Phase III studies in allergy is that the definition of a positive outcome of a Phase III AIT study is different between regulators. For example, the German Paul Erlich Institute (PEI) requires a successful outcome be defined in terms of superiority over placebo using a justified MCID of the primary end-point score defined based on absolute differences. In contrast, the FDA applies a criterion which is based on percentage difference and success defined using a non-inferiority margin with success defined as achieving an upper limit of the 95% confidence interval below −10%.^34^ These FDA efficacy requirements are more stringent for AIT than for small molecule agents, which historically have been licensed using statistical significant differences relative to placebo. It should be noted that these differences can be overcome with selecting the highest sample size when applying both criteria which generally results in large Phase III studies to cover both continents. However, these differences in regulatory perspectives on the definition of success and MCID complicates the global conduct of pivotal Phase III studies for AIT products conducted simultaneously in Europe and United States.

### Placebo effects, blinding conditions in AIT and their influence on phase III study outcomes

The placebo effect is complex and is a mixture of many contributing effects, all of which produce improvements in perceived treatment response through mind-brain influences. Contributors to placebo effect include psychosocial factors, patients expecting to get better when participating in a clinical trial, rituals of care, active engagement in treatment, and the process of taking study medication.^35^ Moreover, the placebo effect may reflect natural disease course or variability in symptoms, regression to the mean, response bias when reporting subjective symptoms and effects of other concurrent treatments. Placebo effects apply to both the placebo and active groups, as the psychologic effect of active patient engagement with the additional attention to patients during regular visits to allergy specialists induces patient improvements independent of the assigned randomized treatment. As long as these placebo effects are independent of the assigned treatment and the therapeutic effect window is large enough, it may be postulated that the comparison between treatments (ie, treatment difference) is not affected by placebo effects. However, the more realistic scenario is that drug-specific effects (eg, from the use of relief/standard of care medications) may interact with the placebo effects to result in a different placebo effect for the active group compared to the placebo group, generally causing a disadvantage for the active treatment under investigation.^36^

Recently, an EAACI position paper was published to provide a better understanding of the placebo effect in AIT.^37^ However, several important areas concerning the use of placebo in current state-of-the-art AIT studies were not discussed. This EAACI task force group stated that *“For SLIT studies, it is accepted that (i) an inert placebo substance cannot adequately mimic the local adverse events induced by an allergen in a substantial proportion of patients, and (ii) ethical considerations prevent the inclusion of histamine in an* ‘*active placebo*’”. The important acknowledgment here is that blinding for SLIT trials cannot be claimed. As recently highlighted by almost the same group of authors, in SLIT trials, placebos with local effects are not available, which makes complete blinding not possible.^38^ In SLIT studies the majority of patients on active treatment experiencing local adverse events in the mouth and throat areas related to the sublingual administration of these products, with oral pruritus, throat irritation, and mouth oedema being most common (Table 3). Experiencing local reactions directly after sublingual administration of verum tablets or drops/puffs causes substantial placebo effects because the mouth area is one of the body's most sensitive parts. Based on the Phase III study results of registered SLIT studies, up to 74% of patients experience mostly local reactions, with incidences 3–4 times higher than those on placebo during the first year of treatment (Table 3). Importantly, the placebo effect is reinforced in the active treatment arm by the *daily* sublingual intake of these products causing repeated local reactions in the mouth area, providing repeated reassurance to patients that they received the active treatment enhancing their feeling of improvement increasing the placebo effect in the active treatment arm. Consequently, an educated guess of the treatment assignment is relatively easy for both patients and investigators. Therefore, SLIT studies are de facto not blinded due to the local reactivity of these products, as supported by literature.^39^ Under such SLIT unblinded conditions, it can be postulated that the placebo effect is more positively affecting the active treatment group than the placebo treatment group. Therefore, it can be assumed that in SLIT Phase III studies the improvements on primary and secondary endpoints of the active treatment versus placebo are over-estimated, caused by the enhanced placebo effect in the active group only. Stated differently, as blinding conditions of SLIT Phase III trials cannot be maintained, especially subjectively measured primary and secondary efficacy results are positively biased towards a more significant improvement compared to placebo. In contrast, for SCIT trials, blinding conditions are much better maintained, local reactivity is experienced in a less sensitive area (arm/shoulder) and mostly induced by a physical injection with a lower dosing frequency (generally 1–4 weeks apart). This difference in placebo response between SLIT and SCIT may have contributed to the more recent higher success rate for SLIT compared to SCIT Phase III studies. Literature evidence indeed underlines that SCIT studies are more severely impacted by placebo response.^17^^,^^43^^,^^44^ Clearly, the positively skewed efficacy results reported by SLIT trials complicates (and possibly invalidates) a direct comparison of treatment effects between Phase III SLIT and SCIT trials.

**Table 3:** Differences between SLIT and placebo in incidences of treatment related adverse events based on pivotal Phase III studies in approved products.

Allergen source	Allergen preparation	Type of AEs	Active (%)	Placebo (%)	Reference
Birch pollen	Drops	Local	59	21	^15^
Grass pollen	Tablet	Related	71 (year 1)	25 (year 1)	^40^
			59 (year 2)	18 (year 2)	
			45 (year 3)	3 (year 3)	
Grass pollen	Tablet	Related	59	24	^35^
Grass pollen	Tablet	Related	73	28	^41^
Grass pollen	Tablet	Related	70	25	^38^
HDM	Tablet	Related	61	16	^17^
HDM	Tablet	Related	51	15	^42^
Birch	Tablet	Related	74	23	^24^
Ragweed	Tablet	Related	up to 69	29	^43^

AEs = adverse events; HDM = house dust mite

The recent cat allergy pivotal Phase III SCIT failure, with placebo effects reaching up to 60%, highlighted the importance of the quantification of the placebo response.^9^ Unfortunately, no baseline season was introduced in any of the recent pollen allergy pivotal Phase III SCIT and SLIT studies and as a consequence, the placebo response on the primary efficacy measure cannot be calculated. However, for field studies evaluating AIT for *perennial allergies* (eg, HDM, cat) during one year, a baseline period can be established by the evaluation of the extent of symptoms and relief medication use during a short period before treatment commences.

### Placebo composition: active or inactive

From a regulatory perspective, the placebo should be a “treatment that appears as identical as possible to the test treatment”.^45^ While this is less challenging for SLIT products from the manufacturing point of view, this is more challenging for SCIT trials due to the injection of a fluid composed of (modified) allergens and adjuvants. As a consequence, the vast majority of modern double-blind placebo-controlled (DBPC) SCIT trials use adjuvants without allergen in the placebo formulation such as aluminium or MicroCrystalline Tyrosine (MCT). The authors of the recently published EAACI position paper focus on the use of histamine as a placebo within SCIT trials, although only very sporadically applied in SCIT.^37^^,^^42^ This is an important consideration as using adjuvants as placebo introduces the concept of “active placebo” versus “inactive placebo” and potential consequences on study outcome. This thought needs to be expanded to “pharmacologically active” versus “immunologically active” placebos and the latter even more to “Th1 polarizing immunologically active” versus “Th2 polarizing immunologically active” placebos. Adjuvants, unlike histamine, activate immunological pathways and aluminium is known to drive a Th2 response whereas other adjuvants like MCT or CaPhos polarize towards a Th1 response. In the context of AIT, this is relevant because AIT induces a shift towards Th1. Hence, using a Th2 polarizing adjuvant like aluminium in SCIT placebo formulations may positively bias the difference between verum and active placebo whereas using a Th1 polarizing active placebo may induce treatment effects in the same direction as the verum does. This may skew the overall treatment effect to be less pronounced than the true treatment effect (ie,. when using inactive placebo). Moreover, adjuvants do create the likelihood of inflammatory responses at the placebo injection sites, which subjects may perceive as being indicative of receiving the active product and therefore heighten placebo effects in immunoadjuvant placebo groups. A study to evaluate this hypothesis is currently completed [EUdraCT 2020-000408-13].

Furthermore, potential safety consequences for the use of active placebo must be considered, especially for SCIT products applying placebo containing metal salts, which accumulate in the body, including the brain, to potentially unacceptable levels in young children.^46^^,^^47^

### Variability in allergen exposure relates to treatment effects and symptom and medication scores

Results of pivotal Phase III seasonal AIT studies in general are considered to strongly depend on the pollen exposure of the particular season the study is conducted. Previously, negative or inconclusive seasonal AIT studies have been attributed to low pollen seasons.^39^^,^^48^ An important success factor for Phase III AIT studies is the accurate planning and site selection based on historic pollen exposures. A post-hoc analysis elegantly showed that for grass SLIT, the treatment effect depends on the extent of grass pollen exposure.^49^ However, this remains an area of further research as this analysis was based on one SLIT grass program only and it is currently unclear whether the results of this post-hoc analysis can be extrapolated to other products and/or other seasonal allergies.

**Fig. 1:**
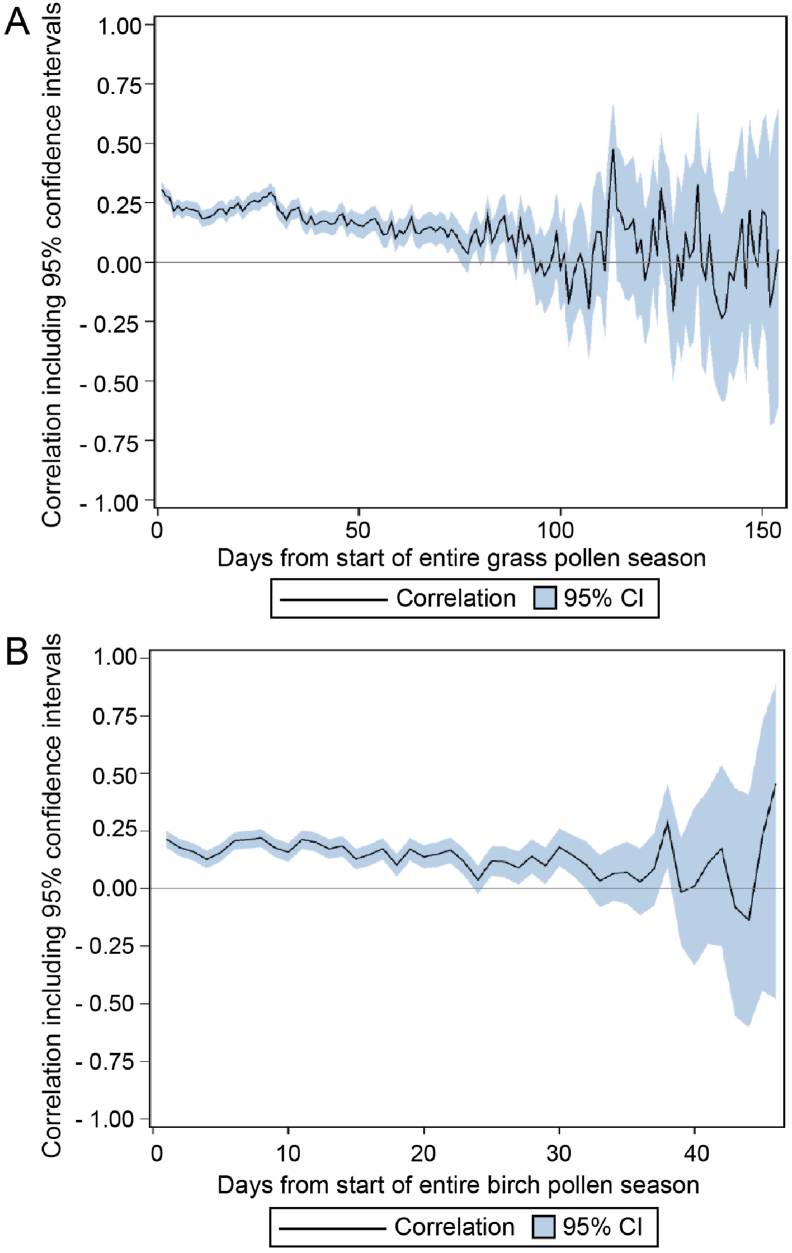
**Correlation between CSMS and grass pollen (A), respectively birch pollen (B) concentrations during the pollen season.** (A) During the first approximately 2 months of the start of the grass pollen season a low but statistically significant correlation was observed between the CSMS and the grass pollen counts. (B) During the first approximately 3 weeks of the start of the birch pollen season a low but statistically significant correlation was observed between the CSMS and the birch pollen counts. (Data originally presented as poster during the EAACI Congress 2018)

Recently, an EAACI expert consensus on definitions of pollen season and peak pollen season for clinical trials of allergen immunotherapy for pollen-induced rhinoconjunctivitis suggest a more unified approach to define end-points in pivotal Phase III studies.^50^ A retrospective evaluation of crowd-sourced symptom data collected in various European countries during 2014–2016 provided initial validation support for the suitability of these definitions for birch and grass pollen allergy.^51^ However, from this latter publication, it was also clear that the correlation between the pollen exposure and field end-point showed a large amount of variability between countries, with even negative correlations for birch in 2015 and correlations <0.10 for grass in 2016 for single European countries. To further evaluate the correlation between the CSMS and the pollen concentrations, the same pollen database was evaluated during an extended period of 2009–2016 using similar methods.^51^ These results confirm a low but statistically significant correlation between grass and birch pollen concentrations and the CSMS (Fig. 1). Importantly, these findings suggest that the optimal window to observe treatment effects after immunotherapy may be a relatively short interval after start of the pollen season and during the peak pollen season.

Reasons for these low correlations between pollen concentrations and symptom and medication load of patients are multi-fold. Notably, the pollen measurements produced are generally measured at substantial heights. Although for some plants more than for others (e.g. grasses) it was verified that symptom data correlate more with rooftop pollen concentrations than with ground pollen concentrations,^52^ it remains questionable if these are representative for the pollen micro-climates of the individual subjects assigned to the same pollen station in the clinical trial (eg, office worker versus gardener). To address this, there are various initiatives to develop individual pollen samplers,^52^ and although this new methodology may offer opportunities for future clinical trials, none are currently validated. Another important reason for these low correlations is that ultimately pollen counting is a crude assessment of actual allergenic protein exposure as pollen fragments are not included in these definitions. It has been demonstrated that pollen concentrations do not always correlate to major allergen content, caused by substantially different amounts of allergens released by the same amount of pollen.^53^

Furthermore, various environmental factors impact the allergen content of pollens and it has also been shown that ozone independently aggravates pollen-induced symptoms.^54^ A study to prospectively evaluate the effect of environmental factors including ozone on the primary CSMS is currently ongoing [EUdraCT 2020-000408-13].

Considering the above, the value of absolute pollen concentrations to define the start and the end of the peak or entire pollen season on an individual patient level is debatable. Despite this, Phase III AIT trials heavily rely on pollen concentration data, and the definition of the peak and the entire pollen season has a direct influence on the primary outcome of these trials.

For successful site selection in Phase III, it is important to produce accurate pollen mapping of the site locations to be included in the study to ensure these sites have sufficient pollen exposure. This requires the availability of historic pollen data over multiple years and use of experienced pollen stations, and preferably organized pollen networks applying qualification procedures of pollen stations and/or centralized readings.^55^ An additional risk to Phase III trials is that during seasons with less pronounced pollen counts, no peak season can be defined based on the EAACI definition, which would exclude a possibly large number of patients from the primary analysis thereby, substantially reducing the power of the study, especially if the peak season is defined as the primary end-point period.

Another important factor for multi-country AIT studies is the selection of the pollen sampler to be used in AIT studies. While in Europe the Burkard pollen sampler is the standard, the most commonly used pollen sampler in the United States is the Rotorod sampler. The Rotorod samples are less suitable for AIT studies as Rotorods do not allow storage of pollen measurements for quality assurance purposes, generally weekend evaluation is not guaranteed due to staff availability, nor can central reading be organized. Currently, there is a new initiative ongoing to establish a Burkard sampler network in the United States, which would allow globally standardized pollen platform and procedures to conduct Phase III AIT studies simultaneously in Europe and the United States.^56^

For Phase III AIT perennial allergen studies, the assessment of allergen exposure is even more challenging than for seasonal allergens. For HDM allergy, the seasonality is difficult to consider and peak exposure strongly depends on the region or country. The collection of house dust samples at home with central measurement is possible, but logistically difficult and costly. These challenges for AIT studies with perennial allergens may have contributed to the limited treatment effects of 16–18% observed in HDM SLIT studies as well as the effect size of only 9.2% reported for a recent pivotal Phase III HDM SCIT study (EudraCT2016-000051-27) as well as the recent Phase III failures of the HDM and cat peptides^9^^,^^11^^,^^13^^,^^17^^,^^20^

### Patient selection and heterogeneity

In AIT studies, the selection criteria for patients participating in AIT Phase III studies are reasonably standardized. Patient selection based on a positive clinical history of moderate to severe symptoms is of vital importance for the success for AIT Phase III studies.^57^ In addition to clinical history, non-allergic rhinitis triggers need to be excluded by a positive skin prick test (SPT) and IgE class ≥2 (ImmunoCAP). Despite applying these standardized criteria, there remains to be a high degree of heterogeneity in study populations. In a recent successful SLIT ragweed paediatric study, subjects with massive levels of allergen specific IgE (>10 kU) were included to enrich the study population and enhance the clinical response. However, such high levels of allergen specific IgE are not typical in sensitized populations and the treatment effects achieved are probably not representative for the general ragweed allergic population.^58^ Recently the use of patient enrichment strategies has been promoted using an objective, standardized nasal or conjunctival provocation or EEC prior to inclusion of subjects with relevant diseases in AIT trials.^24^ A recent post-hoc analysis demonstrated that restricting the patient population to those with a positive CPT result at baseline greatly improved the treatment effect in grass allergy.^7^ Such patient enrichment strategies have recently also been employed in a HDM pivotal Phase III study, where AIT patients were selected based on a CSMS >1.5 and a positive NPT result at baseline, in addition to the standard selection criteria (EudraCT2016-000051-27). However, this study has failed and ironically it was claimed that only approximately 30% of the selected population had moderate to severe symptoms of HDM allergy based on post-hoc analyses, despite employing these additional subject enrichment criteria. Alternatively, EECs could be considered to enhance patient selection, but in a multi-country Phase III study this may be challenging.^24^ Lastly, restrictions on the inclusion of polysensitized participants could be effective in reducing variability and exclusion of patients with moderate to severe clinical manifestation of symptoms caused by multiple other allergen exposures which could impact the primary end-point results due to overlapping evaluation periods should be considered. This was convincingly shown by a recent long-term (5-year) study of the depigmented and glutaraldehyde polymerized allergenic birch extract (EUdraCT 2012-000414-11259). In this study, only for the sub-group of mono-sensitized subjects (40% of the total study population), a statistically significant difference on a primary CSMS from placebo could be demonstrated after 2 and 3 years of treatment (ie, not after 1 year of treatment and treatment-free follow-up years 4 and 5). As no statistically significant different treatment effects were shown for the co-sensitized group, the independent Data Monitoring Committee recommended to withdraw all of the co-sensitized patients from the study and continuing the treatment-free period only with patients mono-sensitized to birch.

### The promise of biomarkers in AIT

The authors believe that there is a strong future role for predictive AIT biomarker footprints to be used as primary end-points and or key secondary end-points in Phase II, possibly also in pivotal Phase III studies. A recent EAACI position paper recommends allergen-specific IgG_4_ as a biomarker for compliance, while the ratio of specific IgE and total IgE and IgE-facilitated allergen binding (IgE-FAB) could be considered as potential surrogate candidates for efficacy.^59^ In recent years novel biomarkers are being used as a predictive tool for AIT efficacy, including IgG, IgG_4_ and IgA in the local target organ (ie, nasal fluid) and cellular biomarkers.^60^

In addition, various studies have focused on exploring the use of cellular biomarkers. These include 1) basophil activation and histamine release, 2) interleukin 10 (IL-10)-producing innate lymphoid cells (ILC10), 3) regulatory dendritic cells (DCreg), 4) type 2 helper T (Th2), Th2A and T follicular helper (Tfh) cells, and 5) regulatory T and B cells (Tregs and Bregs).^59^^,^^61^^,^^62^ Tolerance induction is a hallmark of an effective AIT, and this is characterized by upregulation of Treg and Breg cells. Following AIT treatment, both natural FOXP3^+^ Tregs and inducible Tregs (IL-10^+^, TGF-b^+^ or IL-35^+^) are induced and associated with suppression of Th2 cells.^63^^,^^64^ SCIT to grass pollen has been associated with a decrease in CD4^+^ T cells and Th2 cytokine level in nasal fluid following nasal allergen challenge.^65^ Moreover, allergen-specific Th2 and Th2A cells were found elevated in patients with alder pollen allergy, which is decreased following SCIT therapy. Both SCIT and SLIT could result in the reduction of peripheral Th2 cells, which was associated with clinical symptoms.^66^ Tfh cells are a more novel subset of cells, which have been reported to be similarly affected following AIT.^67^

More recently, the role of IL-10-producing Bregs has been described as one of the mechanisms of tolerance induction following AIT. A study has shown that house dust mite AIT resulted in elevated frequencies of IgA- and IgG_4_-expressing Der p 1-specific B cells, plasmablasts and IL-10^+^ Breg cells,^68^ which significantly correlated with improved clinical symptoms over the course of AIT. Although AIT studies have shown conflicting association with basophil activation, studies using a promising validated assay to measure basophil activation (CD63 and CD203c) and diamine oxidase (DAO) measuring intracellular histamine level showed persistent basophil suppression following AIT.^69^

Furthermore, AIT has been associated with the induction of cellular responses within ILC10, DCregs, Tregs and Bregs. Studies on AIT for grass pollen allergy have consistently shown a reduction in the proportion of circulating ILC2s,^70^ accompanied by the induction of ILC10 which are functional and correlated with clinical symptoms.^71^

More biomarker research is needed to identify and validate predictive biomarkers and biomarker footprints in AIT. It is especially important to correlate these biomarkers to clinical response as part of this validation process. Unfortunately, especially in Phase III, biomarker research is hampered by the absence of an objective and validated primary measure of efficacy for AIT to validate a new biomarker against. Nevertheless, it is essential to include informative biomarkers in pivotal Phase III studies to establish their predictive value of clinical response and use as efficacy markers in AIT to ensure more efficient execution of AIT clinical trials in the future.

## Recommendations and conclusions

Although a successful Phase II clinical study is not a guarantee for success in Phase III, extensive preparation and incorporation of lessons learned from previous successful and unsuccessful pivotal Phase III AIT studies can substantially increase the chance of Phase III success. The recommendations from this evaluation are:

(i) In the case where Phase II studies were performed using a validated provocation test, if feasible, the same test should be included in Phase III. The benefits are three-fold, as it could serve as a justification to counteract the unpredictability of seasonal field study results, it supports the validation of this provocation test for future AIT studies, and it could also improve patient selection for future Phase III studies.
(ii) To justify a MCID, the RQLQ-S offers promise as it has been extensively validated in comparing prior and post-intervention results with a 0.5 point improvement being assessed as clinically relevant.^32^ Hence, it is recommended to incorporate validated quality of life questionnaires (e.g. RQLQ-S) and an informed selection of predictive molecular biomarkers in pivotal Phase III studies. In addition, this would strengthen the justification of the MCID for future studies, and support the validation of the CSMS applied as primary end-point in these studies (and symptom score and medication score separately).
(iii) Apply an informed approach to select the primary endpoint (eg, by including a field end-point in the Phase II study or performing a pilot study before the pivotal Phase III study), justify the MCID in collaboration with regulators and ensure adequate powering of the study using the accepted regulatory criteria.
(iv) Implement a strategy to reduce the placebo effects as much as possible. This includes training of investigators and patients about placebo effects, limiting the visits to essential visits and strategies to enrich the study population with treatment responders.
(v) Accurately plan site selection based on historic pollen data in seasonal AIT studies. Strongly consider measurement of air pollution, especially ozone concentrations and include (spot) sampling for major allergen content as this better guarantees the accurate assessment of start and end of the (peak) pollen season, and allows for correction of treatment effects using statistical approaches. Consider the flexibility of the adaptation of the EAACI criteria for sites where peak seasons are not reached and possibly a methodolgy for correcting the EAACI criteria for the amount of immunologically active allergenic protein in the air. For AIT Phase III studies with perennial allergens, it is mandatory to allow for a baseline evaluation of the primary end-point and it is strongly advised to consider assessment/guarantees/risks of allergen exposure (eg, HDM sampling or cat cafés).
(vi) Incorporate efficient patient selection strategies, consider methods for patient enrichment (eg, based on the outcome of provocation test or exposure chamber results) to better ensure the selection of an adequate population of patients with moderate to severe allergy and optimize the treatment effect estimate. Remain persistent after a negative Phase III result as even one of the first registered SLIT products suffered from a negative pivotal Phase III study.^49^

In conclusion, these authors strongly advocate innovative well-designed AIT field studies to increase the common understanding of the placebo response, specifically in an AIT Phase III study setting. Such results would provide further guidance for improvements of Phase III clinical study designs, important validation insights of pivotal Phase III primary end-points and biomarker signatures for various allergies, better blinding procedures and improved recommendations on the use of inactive placebo groups in SCIT trials. The latter is especially important to reduce potential safety risks in vulnerable populations (eg, paediatrics) associated with active placebo formulations containing metal salts. Finally, the non-blinding conditions of SLIT studies constitute a significant concern, as the experience of local oral symptoms of these products reveal the treatment assignment and tends to amplify the placebo effect for the active treatment only. This in turn could trigger a positive bias to the outcomes of especially the subjective primary and secondary outcomes of pivotal Phase III studies, which downgrades the level of clinical efficacy evidence of SLIT products and warrants more attention from both allergy practitioners and regulatory agencies.

## Abbreviations

AIT: allergen immunotherapy, Breg: regulatory B cells, CPT: conjunctival provocation test, CSMS: combined symptom and medication score, DAO: diamine oxidase, DBPC: double-blind placebo-controlled, DC: dendritic cells, DCreg: regulatory dendritic cells, EAACI: European Academy of Allergy and Clinical Immunology, EEC: environmental exposure chambers, EMA: European Medicine Agency, FDA: Food and Drug Administration, HDM: house dust mite, Ig: Immunoglobulin, IgE-FAB: IgE-facilitated allergen binding, IL: interleukin, ILC: innate lymphoid cells, i.m.: intramuscular, MA: marketing authorization, MAA: marketing authorization application, MCID: minimal clinical important difference, MCT: microcrystalline tyrosine, MPLA: monophosphoryl lipid A, NPP: Named Patient Products, NPT: nasal provocation test, PEI: Paul Ehrlich Institut, PDCO: paediatric committee, ROC: receiver operating characteristic, RQLQ-S: Standardized rhinitis quality of life score, s.c.: subcutaneous, SCIT: subcutaneous immunotherapy, SLIT: sublingual immunotherapy, SPT: skin prick test, TAO: Therapy Allergen Ordinance, TCS: total combined score, Tfh: T follicular helper, Th2: type 2 helper T, Treg: regulatory T cells, TNSS: total nasal symptom score, WAO: World Allergy Organisation.

## Funding

Allergy Therapeutics (UK) Ltd. sponsored the article publishing fee.

## Author contributions

PJDK, MFK and MHS provided substantial input in the form of writing parts of this manuscript. The remaining authors all supported a constructive and intensive review during several versions of the manuscript leading to this final version.

## Ethics approval and consent to participate

Not applicable.

## Consent for publication

The authors provide their consent for the publication of the manuscript.

## Data availability

Not applicable.

## Declaration of competing interest

PJDK, MFK, KO, MDH, AG, FS and MAS are employees of Allergy Therapeutics Plc / Bencard Allergie GmbH. EJJ reports and Inventor on EP2894478; “LCN2 as a tool for allergy diagnostic and therapy”, EP 14150965.3, Year: 01/2014; US 14/204,570, owned by Biomedical International 458 R+D GmbH, Vienna, Austria, where she is shareholder. She received honoraries for lectures from Bencard Allergy AG, Germany and Allergy Therapeutics, UK, Vifor Pharma, Meda, Novartis, Sanofi. SZ reports grants and personal fees from Allergy Therapeutics, during the conduct of the study; grants and personal fees from bene-Arzneimittel GmbH, grants from ALK Arzneimittel, personal fees from Novartis GmbH, Böhringer Ingelheim, Lofarma GmbH, IMS HEALTH GmbH & Co. OHG, GSK, Stallergenes, Procter and Gamble, Allergopharma GmbH, AstraZeneca, Sanofi/Pasteur, and Aimmune, outside the submitted work. CV reports personal fees from Allergy Therapeutics, Bencard Allergie, grants and personal fees from Allergopharma, personal fees from HAL Allergy, Stallergenes Greer, Novartis Pharma, LETI Pharma, grants and personal fees from DBV Technology, personal fees from Aimmune, Sanofi Aventis outside the submitted work. PZ reports grants and personal fees from ALK Abello, personal fees from Allergopharma, Bencard, HAL, LETI, Meda, grants and personal fees from Marinomed, personal fees from Merck, Novartis, Sigmapharm, Stallergenes, ThermoFisher Scientific outside the submitted work. RM reports personal fees from ALK, grants from ASIT biotech, personal fees from Allergopharma, Allergy Therapeutics, grants and personal fees from Bencard, grants from Leti, grants, personal fees and non-financial support from Lofarma, non-financial support from Roxall, grants and personal fees from Stallergenes, grants from Optima, personal fees from Friulchem, personal fees from Hexal, Servier, Klosterfrau, non-financial support from Atmos, personal fees from Bayer, non-financial support from Bionorica, personal fees from FAES, GSK, MSD, Johnson&Johnson, Meda, personal fees and non-financial support from 10.13039/100004336Novartis, non-financial support from Otonomy, personal fees from Stada, and UCB, non-financial support from Ferrero, grants from BitopAG, Hulka, personal fees from Nuvo, grants from Ursapharm, personal fees from Menarini, Mundipharma, Pohl-Boskamp, grants from Inmunotek outside the submitted work. LK reports grants and personal fees from Allergopharma, MEDA/Mylan, personal fees from HAL Allergie, grants from ALK Abelló, grants and personal fees from LETI Pharma, grants from Stallergenes, and Quintiles, grants and personal fees from Sanofi, grants from ASIT biotech, and Lofarma, personal fees from Allergy Therapeutics, grants from AstraZeneca, GSK, Immunotek, personal fees from Cassella med outside the submitted work; and Membership: AeDA, DGHNO, Deutsche Akademie für Allergologie und klinische Immunologie, HNO-BV, GPA and EAACI. LMD reports personal fees from Allergy Therapuetics, ALK Abello, AstraZeneca, SanofiGenzyme, Regeneron, GSK, Abionic, outside the submitted work. WGS reports personal fees from Allergy Therapeutics, ALK, Aimmune, FARE, other from DOTS, personal fees from GSK, Novartis, Regeneron, grants and personal fees from Sanofi, outside the submitted work. JAB reports grants and personal fees from Allergy Therapeutics, personal fees from ALK, during the conduct of the study. TMK is under consultancy agreements with Allergy Therapeutics, and is co-founder of Saiba GmbH. MHS, MHB, and UB have nothing to disclose.
